# Lavender Decline in France Is Associated with Chronic Infection by Lavender-Specific Strains of “Candidatus Phytoplasma solani”

**DOI:** 10.1128/AEM.01507-18

**Published:** 2018-11-30

**Authors:** Olivier Sémétey, Jonathan Gaudin, Jean-Luc Danet, Pascal Salar, Sébastien Theil, Marie Fontaine, Michel Krausz, Eric Chaisse, Sandrine Eveillard, Eric Verdin, Xavier Foissac

**Affiliations:** a Conseil Interprofessionnel des Huiles Essentielles Françaises (CIHEF), Manosque, France; b UMR1332 Biologie du Fruit et Pathologie, INRA, Université de Bordeaux, CS20032, Villenave d'Ornon, France; c Centre Régionalisé Interprofessionnel d'Expérimentation en Plantes à Parfum, Aromatiques et Médicinales (CRIEPPAM), Manosque, France; d UR0407 Unité de Recherche de Pathologie Végétale, INRA, CS60094, Montfavet, France; University of California, Davis

**Keywords:** *Hyalesthes obsoletus*, *Hymenobacter*, molecular epidemiology, *Phytoplasma*, planthopper, stolbur, genotyping, phloem-limited bacteria

## Abstract

Lavender decline compromises French lavender production, and preliminary data have suggested the involvement of “Candidatus Phytoplasma solani” in the etiology of the disease. In order to evaluate the epidemiological role of “*Ca*. Phytoplasma solani,” a 3-year survey was conducted in southeastern France. “*Ca*. Phytoplasma solani” was detected in 19 to 56% of the declining plants, depending on seasons and cultivars, and its prevalence was correlated with symptom severity. Autumn was more favorable than spring for phytoplasma detection, and “*Ca*. Phytoplasma solani” incidence was higher in Lavandula angustifolia than in Lavandula intermedia hybrids. Detection of the phytoplasma fluctuated over months, supporting the chronicity of infection. Three “*Ca*. Phytoplasma solani” *secY* genotypes, S17, S16, and S14, were the most prevalent in lavender fields and were also detected in nurseries, whereas strains detected in surrounding bindweed and wild carrots were mostly of the S1 and S4 genotypes. This suggests that lavender is the main pathogen reservoir of the epidemic. Adults and nymphs of the planthopper vector Hyalesthes obsoletus were commonly captured in lavender fields and were shown to harbor mainly the prevalent phytoplasma genotypes detected in lavenders. The “*Ca*. Phytoplasma solani” genotype S17 was transmitted to Catharanthus roseus periwinkle by naturally infected H. obsoletus. Finally, the inventory of the bacterial community of declining lavenders that tested negative for “*Ca*. Phytoplasma solani” by 16S rRNA deep sequencing ruled out the involvement of other phloem-limited bacterial pathogens.

**IMPORTANCE** The etiology and main pathways for the spread of lavender decline, an infectious disease affecting French lavender production since the 1960s, have remained unclear, hampering the development of efficient control strategies. An extensive survey of lavender fields led to the conclusion that “Candidatus Phytoplasma solani” was chronically infecting declining lavenders and was associated with large infectious populations of Hyalesthes obsoletus planthoppers living on the crop itself. Lavender appeared to be the main reservoir host for lavender-specific phytoplasma strains, an unusual feature for this phytoplasma, which usually propagates from reservoir weeds to various economically important crops. These results point out the necessity to protect young lavender fields from the initial phytoplasma inoculum coming from surrounding lavender fields or from infected nurseries and to promote agricultural practices that reduce the development of H. obsoletus vector populations.

## Abstract (precis)

The etiology and main pathways for the spread of lavender decline, an infectious disease affecting French lavender production since the 1960s, have remained unclear, hampering the development of efficient control strategies. An extensive survey of lavender fields led to the conclusion that “Candidatus Phytoplasma solani” was chronically infecting declining lavenders and was associated with large infectious populations of Hyalesthes obsoletus planthoppers living on the crop itself. Lavender appeared to be the main reservoir host for lavender-specific phytoplasma strains, an unusual feature for this phytoplasma, which usually propagates from reservoir weeds to various economically important crops. These results point out the necessity to protect young lavender fields from the initial phytoplasma inoculum coming from surrounding lavender fields or from infected nurseries and to promote agricultural practices that reduce the development of H. obsoletus vector populations.

## INTRODUCTION

Lavender is cultivated in 20,000 ha in southeastern France at altitudes of 600 to 1,200 m. Lavandula angustifolia, the noble lavender, represents only 20% of the production, as the cultivation of Lavandula intermedia clones, i.e., hybrids of L. angustifolia and Lavandula latifolia, has continuously increased since the 1950s. Lavender decline (LD) has affected lavender production in France since the late 1960s, initially affecting the L. intermedia cultivar Abrial. A phytoplasma was observed in the phloem of declining Abrial by electron microcopy and transmitted to Catharanthus roseus using Cuscuta subinclusa (dodder plant) (1). Later, “Candidatus Phytoplasma solani,” formerly known as stolbur phytoplasma (2), could be detected in declining lavenders maintained *in vitro* (3). “*Ca*. Phytoplasma solani” is endemic to Europe and is the agent of various crop diseases such as stolbur of solanaceous crop, bois noir disease of grapevine, yellowing of strawberry, and maize redness (2, 4–7). The main vector in Western Europe is the Cixiidae planthopper Hyalesthes obsoletus, which propagates the phytoplasma from infected weeds, such as bindweed and stinging nettles, on which the insect develops its life cycle (8–11). Abundant larva populations of H. obsoletus had been observed on declining lavenders, and H. obsoletus was shown to be able to complete its life cycle on lavender (12, 13).

LD epidemics have recently been a focus of attention due to the increasing damages that the disease has steadily provoked since the 2000s and because LD recently spread to cultivars thought to be tolerant to the disease. Detection of “*Ca*. Phytoplasma solani” by classical nested PCR had presumably been hampered by the presence of PCR inhibitors in lavender extracts, and therefore the involvement of “*Ca*. Phytoplasma solani” as the main causal agent of LD epidemics could not be demonstrated. A real-time PCR assay for the detection of “*Ca*. Phytoplasma solani” in woody hosts was less sensitive to PCR inhibitors (14) and made possible the detection of “*Ca*. Phytoplasma solani” in lavender. To evaluate the involvement of “*Ca*. Phytoplasma solani” in the disease, 27 fields in southeastern France were surveyed for LD from 2008 to 2010. A specific real-time PCR assay was used to assess the association of “*Ca*. Phytoplasma solani” with LD and monitor the kinetics of lavender infection. To trace the spread of phytoplasma strains, the *secY* genotype of “*Ca*. Phytoplasma solani” strains detected in declining lavenders and in H. obsoletus populations was determined and compared to that of the phytoplasma strains detected in H. obsoletus, in nurseries, and in infected weeds collected in the surroundings of lavender fields. Finally, as LD could involve other phloem-limited bacteria, bacterial communities of phytoplasma-free healthy and declining lavenders were compared through deep sequencing of 16S rRNA.

## RESULTS

### Incidence of lavender decline and prevalence of “*Ca*. Phytoplasma solani” infection in declining lavenders from production fields and nurseries.

**FIG 1:**
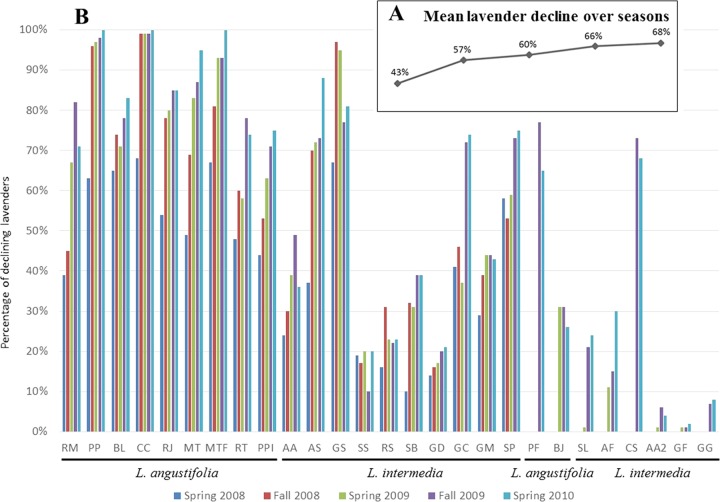
Temporal evolution of lavender decline incidence. (A) Mean lavender decline calculated for 19 fields surveyed from spring 2008 to spring 2010. (B) Incidence of lavender decline in each surveyed field. The lavender field code is indicated below the graph, and field characteristics are given in Table 4. Lavender fields are sorted according to cultivars, and fields on the left are those added to the survey to replace fields pulled out in 2008 and 2009.

**FIG 2:**
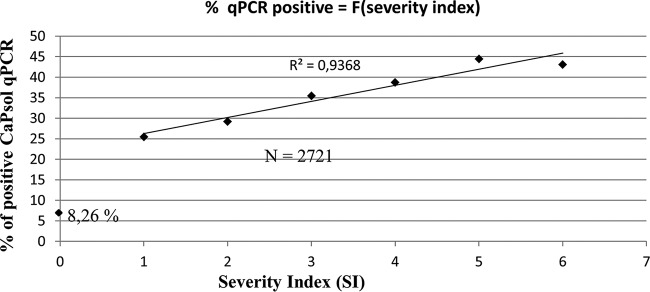
Positive linear correlation between real-time PCR detection of “Candidatus Phytoplasma solani” and lavender decline severity index (SI). The coefficient of determination (*R*^2^) corresponds to the best fitted line excluding healthy carriers (SI = 0). N corresponds to the number of lavender samples tested.

At the beginning of the survey, LD affected 43% of the lavenders (Fig. 1A) and its prevalence ranged from 10% in L. intermedia field SB to 68% in *L. angustifolia* field CC (Fig. 1B). The initial incidence of the disease was significantly higher in L. angustifolia fields, 52.3% on average, than in L. intermedia fields, in which the average LD incidence was 31.5%. During the 3 years of the survey, the disease progressed in all fields except in the L. intermedia Sumian field SS (Fig. 1B). When the survey ended in spring 2010, some of the fields had been pulled out due to the high incidence of the disease, and LD was affecting 68% of the lavenders (Fig. 1A) and on average 76% of L. angustifolia and 53.5% of L. intermedia plants. To evaluate the association of “*Ca*. Phytoplasma solani” with declining lavenders, 15 declining and 4 symptomless lavenders were randomly sampled in each field at the end of spring and beginning of autumn 2008 and 2009, and their total nucleic acids were subjected to “*Ca*. Phytoplasma solani”-specific real-time PCR. A total of 1,970 diseased plants were tested; 37.1% gave a positive detection test result (Table 1). “*Ca*. Phytoplasma solani” detection was significantly higher in autumn in both L. angustifolia and L. intermedia fields than in the spring and was significantly higher for L. angustifolia than for L. intermedia (Table 1). The maximum detection rate was 56.1% in declining L. angustifolia when tested in autumn. A linear correlation was observed between the percentage of “*Ca*. Phytoplasma solani”-positive lavenders and the severity index (SI) (Fig. 2). While “*Ca*. Phytoplasma solani” was detected in 25 to 30% of declining lavenders with low SIs of 1 to 2, the detection peaked at 43 to 45% in severely affected lavenders with SIs of 5 to 6. It must be noted that 8.26% of symptomless lavenders were positive for “*Ca*. Phytoplasma solani” detection. These nonsymptomatic carriers were equally distributed among L. angustifolia and L. intermedia. They were found in all cultivars and clones except in L. angustifolia Matherone and C15-50, which had, however, been sampled only in the two highly declining fields MTF and CC, respectively (Fig. 1).

**TABLE 1:** Incidence of “*Ca*. Phytoplasma solani”-positive plants in declining plants according to seasons and lavender species from 2008 to 2010

Season or species	Total no. of plants tested	No. of “*Ca*. Phytoplasma solani”-positive plants	% of “*Ca*. Phytoplasma solani”-positive plants	Statistical significance (chi-square test)
Season				8.3 × 10^−10^
Spring	1,023	297	29.0	
Autumn	947	435	45.9	
Species				4.0 × 10^−10^
L. angustifolia	869	407	46.8 (39.7–56.1)^*a*^	
L. angustifolia × L. latifolia hybrids (L. intermedia)	1,101	325	29.5 (19.3–39.1)^*a*^	

a Range in parentheses indicates the values for spring and autumn.

Of a total of 3,056 lavender samples collected in nurseries, 74 were positive for “*Ca*. Phytoplasma solani” (2.4%), most of which were grown in open fields (see Table S1 in the supplemental material). Only 4 lavenders propagated from certified mother stocks in insect-proof tunnels were positive; those were collected at the same nursery.

### Time course evolution of “*Ca*. Phytoplasma solani” infection and disease spread.

**FIG 3:**
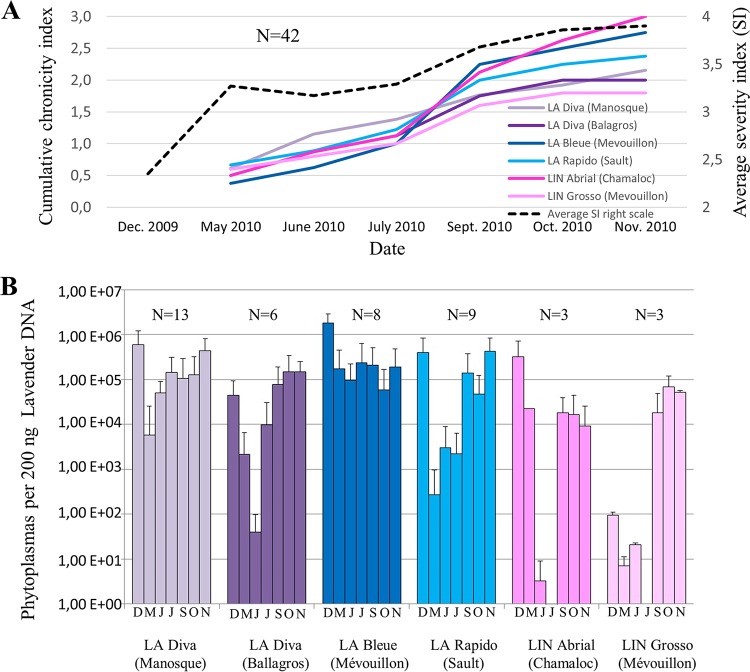
Chronicity of “Candidatus Phytoplasma solani” detection and phytoplasma titer in positive lavenders. Locations of lavender fields of Lavandula angustifolia (LA) and L. hybrida (LIN) are indicated in parentheses. N corresponds to the number of lavender samples tested. (A) Mean severity index (dotted line, right scale) and cumulative proportion of lavender having changed phytoplasma detection status in each field (full colored lines, left scale). (B) Mean titers in phytoplasma-positive lavenders as determined by quantitative PCR. Months of sampling are indicated below graphs.

The difference of prevalence between spring and autumn could result from the chronicity of the infection and/or the fluctuation in phytoplasma population. To assess the influence of these two parameters, we carried out a time course survey of phytoplasma infection in lavenders. In November and December 2009, 42 lavenders positive for “*Ca*. Phytoplasma solani” detection were selected in 4 L. angustifolia and 2 L. intermedia fields located in different lavender production areas. The following year, lavenders were scored monthly on a severity index and sampled for detection and quantification of “*Ca*. Phytoplasma solani.” The average severity index increased from 2.35 in December 2009 to 3.9 in November 2010 (Fig. 3A). In May 2010, “*Ca*. Phytoplasma solani” was detected in only one-half of the lavenders previously determined to be positive for “*Ca*. Phytoplasma solani.” The detection status fluctuated in all fields, and the cumulative number of lavenders that changed detection status, here named cumulative chronicity index, increased over the year (Fig. 3A). In November 2010, lavenders had changed detection status on average from 1.8 times for the L. intermedia clone Grosso in Mévouillon to 3 times for the L. intermedia clone Abrial in Chamaloc. Only 5 L. angustifolia plants stayed positive for “*Ca*. Phytoplasma solani” throughout the survey. In addition to the chronicity of “*Ca*. Phytoplasma solani” infection, the phytoplasma population detected in lavender fluctuated over the year. In all fields, the population was lower in spring 2010 than that previously measured in November and December 2009 (Fig. 3B). However, the phytoplasma population increased during summer and again reached a maximum during autumn. The phytoplasma population was high and fluctuated poorly in the highly susceptible L. angustifolia cultivar Bleue, even though the chronicity of the infection was also high.

To evaluate disease spread, all declining and nonsymptomatic lavenders that tested negative for “*Ca*. Phytoplasma solani” in 2008 and 2009 were sampled and tested again in 2010 (see Table S2 in the supplemental material). The incidence of “*Ca*. Phytoplasma solani” increased in all fields surveyed but more markedly in L. angustifolia than in L. intermedia fields. As expected, the increase was higher when the lavenders had been exposed to two summer seasons, when inoculation by the insect vectors takes place.

### Monitoring of H. obsoletus population levels and infectivity.

**FIG 4:**
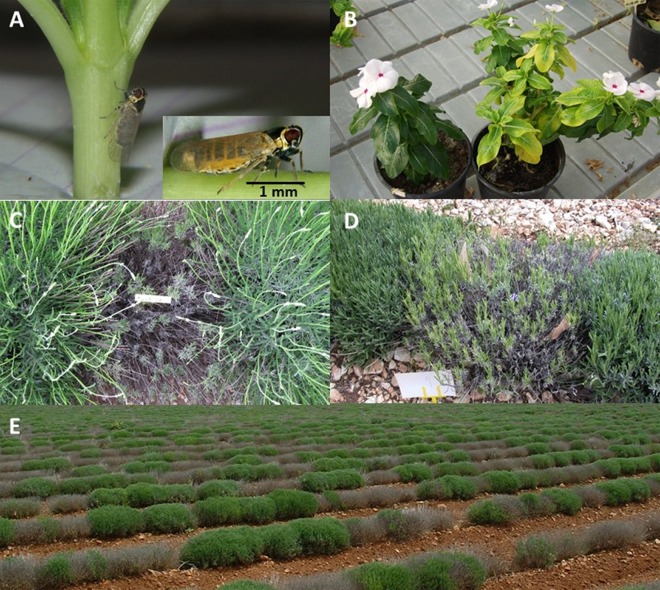
Experimental transmission with naturally infected Hyalesthes obsoletus planthoppers and disease symptoms. (A and B) Naturally infected H. obsoletus in transmission assay (A), symptoms induced on Catharanthus roseus Madagascar periwinkle by “Candidatus Phytoplasma solani” strain Champlong of *secY* genotype S17 (B, right) and healthy C. roseus control (B, left). (C and D) Lavandula angustifolia cultivar Rapido (C) and L. intermedia cultivar Super (D) infected by “*Ca*. Phytoplasma solani” strain of genotype S17. (E) A 4-year-old field of L. angustifolia cultivar C15-50 where genotypes S17 and S14 represent 45% and 27% of the disease cases.

In the spring of 2008, roots of lavenders were examined for the presence of nymphs by uprooting 10 plants in each field. The presence of the young insects was irregular but slightly more frequent and abundant on L. angustifolia than on L. intermedia root systems (Table 2). The difference was more significant in summer populations, with large populations of 148 and 423 H. obsoletus adults collected on L. angustifolia fields PP (Fine) and BM (Bleue), respectively, and the absence of H. obsoletus adults on L. intermedia. In 2008, 8 to 20% of the H. obsoletus adults tested positive for “*Ca*. Phytoplasma solani.” H. obsoletus insects captured on L. angustifolia in field PP were caged on C. roseus seedlings for transmission assays. Two months later, one of the four periwinkle plants exposed to insects exhibited leaf yellowing and tested positive for “*Ca*. Phytoplasma solani”; this plant is referred to as strain Champlong (Fig. 4A and B). In 2009, nymphs were not monitored but large populations of 213 and 293 H. obsoletus adults were again collected on L. angustifolia Fine (PF) and Bleue (BJ) fields, respectively. In contrast to year 2008, 6 to 119 H. obsoletus adults were captured on L. intermedia of clones Super, Grosso, and Abrial. The “*Ca*. Phytoplasma solani”-infected insects represented 6 to 66% of the various H. obsoletus populations.

**TABLE 2:** Populations of H. obsoletus collected in lavender fields and “*Ca*. Phytoplasma solani” real-time PCR-positive adults

Field code	Cultivar	Yr	No. of nymphs^*a*^	No. of adults^*b*^	% PCR positive
BM	L. angustifolia Bleue	2008	34	423	8
PP	L. angustifolia Fine	2008	54	148	20
RM	L. angustifolia Maillette	2008	0	1	ND^*c*^
AF	L. intermedia Abrial	2008	15	0	ND
RS	L. intermedia Super	2008	0	0	ND
GC	L. intermedia Grosso	2008	15	0	ND
GS	L. intermedia Grosso	2008	0	0	ND
SS	L. intermedia Sumian	2008	0	0	ND
SP	L. intermedia Super	2009	ND	6	66
AA	L. intermedia Abrial	2009	ND	119	65
PF	L. angustifolia Fine	2009	ND	213	35
SB	L. intermedia Super	2009	ND	98	6
BJ	L. angustifolia Bleue	2009	ND	293	30
GC	L. intermedia Grosso	2009	ND	111	42

a For 10 plants examined.

b For 15 min D-Vac aspiration.

c ND, not done.

### Genotyping of “*Ca*. Phytoplasma solani” strains in declining lavenders from lavender fields and nurseries.

**FIG 5:**
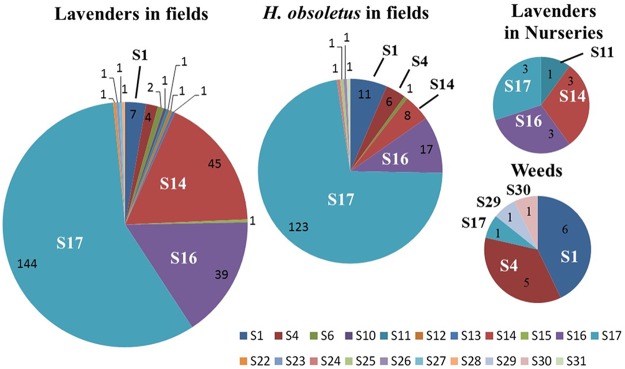
Prevalence of “Candidatus Phytoplasma solani” *secY* genotypes in the lavender agrosystem. Numbers of cases are indicated in each pie chart sector.

The “*Ca*. Phytoplasma solani” strains infecting lavenders were genetically characterized by sequencing the housekeeping gene *secY*. A total of 15 different genotypes were detected in lavender fields and nurseries, of which only 3, the common genotypes endemic to Europe, S1, S4, and S6, had previously been detected in Europe (see Fig. S1 in the supplemental material). None of the 15 genotypes clustered with genotypes S6/S7/S20, for which the main known wild plant reservoir is the stinging nettle Urtica dioica. Most of the new lavender genotypes were one to few single nucleotide polymorphism (SNP) variants of the common genotypes S1 (9 genotypes) and S4 (2 genotypes). The abundant genotype S14 corresponded to the old “*Ca*. Phytoplasma solani” strain Dep transmitted from lavender to C. roseus periwinkle (1). The genotype S16 was clearly branching apart and was significantly different from the other genotypes reported. Three genotypes, namely, S17, S14, and S16, had the highest prevalence in production fields. They respectively represented 57.9%, 17.7%, and 16.1% of the 254 infected lavenders tested, while the common European genotypes S1, S4, and S6 represented only 2.8%, 1.6%, and 0.8%, respectively (Fig. 5). The other nine genotypes were detected only once. The three main genotypes were widely distributed in the lavender fields surveyed (see Table S3 in the supplemental material). Symptoms observed on lavenders infected by genotypes S17 are shown on Fig. 4C to E. S17 was present in all of the 22 fields, and S14 and S16 were detected in 15 and 14 fields, respectively. In addition, the prevalence of the main genotypes varied over the seasons (Table 3). The prevalence of S17 increased from 30.3% in spring 2008 to reach 63.4% of the cases in 2009, while the sum of the prevalence of S16 and S14 fluctuated from 21.2% to 39.3% depending on the year and on the season. In comparison, the other “*Ca*. Phytoplasma solani” genotypes, which constituted 48.5% of the cases at the beginning of the survey in 2008, were barely detected in autumn 2009 (Table 3). In lavender nurseries, the situation was similar to that of production fields; the S17, S14, and S16 genotypes were, however, detected in equal numbers, and S11 was detected in only one plant.

**TABLE 3:** Prevalence of the major “*Ca*. Phytoplasma solani” *secY* genotypes in diseased lavender and L. intermedia in different seasons

Main genotype	Prevalence (% of cases)			
	Spring 2008	Autumn 2008	Spring 2009	Autumn 2009
S17	30.3	50	63.9	63.4
S16	3	14.3	19.7	17.4
S14	18.2	25	16.4	13.7
S1	18.2	3.6	0	0.9
S4	3	1.8	0	4.6
S6	6	0	0	0
Other	21.3	5.3	0	0

**FIG 6:**
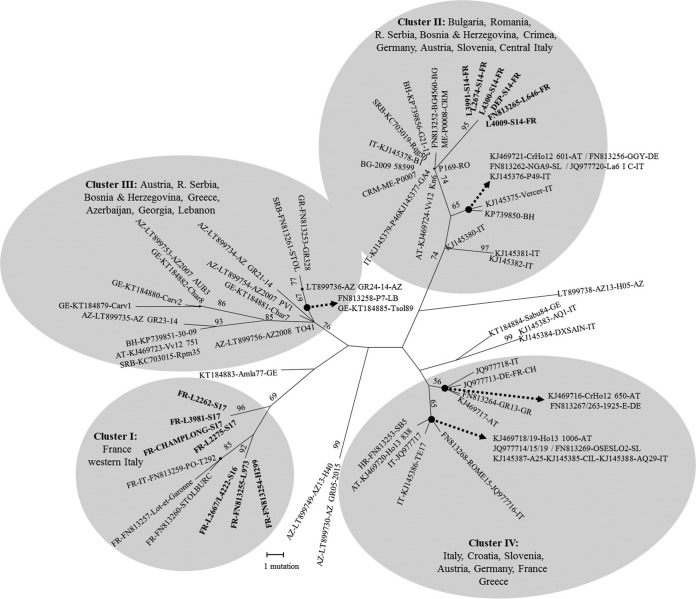
Genetic characterization of “Candidatus Phytoplasma solani” strains detected in lavender fields according to the STAMP gene. Maximum parsimony analysis of “*Ca*. Phytoplasma solani” STAMP gene sequences reported in the Euro-Mediterranean basin. The numbering of genetic clusters is according to Fabre et al. (23). One of the two parsimonious trees is presented. The percentages of replicate trees in which the associated taxa clustered together in the bootstrap test (250 replicates) are shown next to the branches. All positions containing gaps and missing data were eliminated. There were a total of 465 positions in the final data set.

The three main genotypes detected in lavenders were further characterized, as well as the two “*Ca*. Phytoplasma solani” reference strains from lavenders maintained in C. roseus, Dep (1) and Champlong. The *tuf secY vmp1* STAMP gene multilocus sequence analysis evidenced three sequence types in lavender isolates. According to previously published classifications (15–18), they classified as tuf-b1/S17/V4/ST20, corresponding to the strain Champlong, tuf-b2/S14/V1/ST10, corresponding to strain Dep, and tuf-b1/S16/V4/ST5, not yet transmitted to C. roseus (see Table S4 in the supplemental material). The 3 STAMP gene genotypes ST5, ST10, and ST20 have never been detected so far in other crops or in other countries, but phylogenetic analysis of all known STAMP gene sequences (see Table S6 in the supplemental material) showed that genotypes ST5 and ST20, corresponding to *secY* genotypes S16 and S17, respectively, clustered with French strains of “*Ca*. Phytoplasma solani,” while genotype ST10 (reference isolate Dep of *secY* genotype S14) clustered with “*Ca*. Phytoplasma solani” strains detected in grapevine in Southeastern Europe (Fig. 6).

### Prevalence of “*Ca*. Phytoplasma solani” genotypes in H. obsoletus populations and weeds.

The insect vector H. obsoletus is known to reproduce on lavender but also on Convolvulus arvensis bindweeds, which are frequent within lavender fields and their surroundings. Adults of H. obsoletus multiply and transmit the phytoplasma strains that they have acquired at the nymphal stage during the 9 months they spend feeding on the root system of their host plant. Even though nymphs were found on the root system of lavenders, the identity of the “*Ca*. Phytoplasma solani” genotypes found in insects, if compared to that detected in lavender and weeds, could confirm the plant origin of the adults and indicate the plant source of the inoculum. All “*Ca*. Phytoplasma solani” strains detected in insects and collected in the 7 lavender fields monitored in 2008 and 2009 were therefore submitted to *secY* genotyping. Genotype S17 was detected in 72.3% of infected H. obsoletus adults, and genotypes S16 and S14 occurred in 10% and 4.7%, respectively, of the infected insects (Fig. 5).

Weeds growing within the fields surveyed for insects and in their surroundings were surveyed for yellowing or declining symptoms. Among 245 weed plants tested for “*Ca*. Phytoplasma solani” over 3 years, only 42 plants gave positive real-time PCR signals. Only 14 plants with real-time cycle threshold (*C_T_*) values lower than 35 yielded *secY* amplicons that could be sequenced (see Table S5 in the supplemental material). Genotype S1 was detected in 6 plants, mainly in the bindweeds C. arvensis and Convolvulus siculus, and in a wild blackberry, Rubus fruticosus. Genotype S4 was detected in 3 C. arvensis plants, one wild carrot (Daucus carota), and a wild lavender. S17 was found in one C. arvensis plant, and two new *secY* genotypes, S29 and S30, were detected in two unidentified weeds (Fig. 5 and Table S5). Therefore, in contrast to lavenders and H. obsoletus, which harbored mostly genotypes S17, S16, and S14, weeds were infected mainly with S1 and S4 genotypes.

### Detection of other phytoplasmas and comparison of bacterial microbiomes in declining and healthy lavenders negative for “*Ca*. Phytoplasma solani.”

The possible involvement of other phytoplasmas in the etiology of lavender decline was assessed by testing “*Ca*. Phytoplasma solani”-negative declining lavenders sampled in autumn 2008 from fields GD, MTF, GS, RS, and PPi with hypersensitive universal 16S rRNA nested PCR for phytoplasmas. Of 71 declining lavenders tested, one L. angustifolia cv. Matherone plant of field MTF and one L. intermedia of cv. Grosso plant from field GS gave a positive 16S rRNA amplification, whereas 20 symptomless lavenders and 5 greenhouse-grown lavender seedlings tested negative. In both of the positive cases, the sequences were 100% identical to the 16S rRNA sequence of “Candidatus Phytoplasma trifolii” (GenBank accession number AY390261) (19). Two “Candidatus Phytoplasma solani”-positive L. intermedia cv. Grosso plants from field RS (*C_T_* values of 28.6 and 32.5) gave 16S rRNA amplicons with sequence similarity of 99.7% to “*Ca*. Phytoplasma solani” 16S rRNA (GenBank accession number AF248959) (2).

To search for other bacteria associated with declining lavenders, the bacterial community present on healthy lavenders was compared to that of “*Ca*. Phytoplasma solani”-negative declining lavenders. For that purpose, the V4V5 region of prokaryotic 16S rRNAs was amplified from pools of total nucleic acids of nonsymptomatic (NS) and declining lavenders (S). Deep Solexa sequencing produced reads that were clustered to form operational taxonomic units (OTUs). Among the 1,689 OTUs generated, 11 corresponded to mitochondrial and 14 to chloroplastic 16S rRNA regions. These OTUs accounted for 346,814 reads in the case of pool NS and 412,233 reads in the case of pool S, showing an increase of 18.9%, logically reflecting the difference in numbers of sequence reads generated for pool S, which was higher by 19.5%. The number of other prokaryotic reads was 21.1% higher for pool S (38,441 reads) than for pool NS (30,309 reads). This value was used to adjust the number of reads in OTUs of pool S, and the increase of OTU prevalence between pools S and NS was compared (see Fig. S2 in the supplemental material). Two OTUs corresponding to “*Ca*. Phytoplasma solani” accounted for 6 reads in pool S and 2 reads in pool NS. Five OTUs corresponded to phytoplasmas of the taxonomic subgroups 16SrV-C and 16SrV-D but were overrepresented in pool NS (27 reads) in comparison to pool S (15 reads, adjusted value). Finally, 4 reads corresponding to “Candidatus Phlomobacter fragariae” OTUs were detected only in pool S, and no OTU corresponded to “Candidatus Liberibacter” species.

OTUs present as traces and corresponding to two symbionts of H. obsoletus were overrepresented in pool S, certainly reflecting more-frequent contact of these lavenders with this insect. Similarly, OTUs corresponding to mealybug symbionts were overrepresented. Overrepresented OTUs from environmental bacteria included Luteolibacter sp. present as traces, soil-born Actinomycetales, and Hymenobacter sp. Remarkably, OTUs from Hymenobacter sp. were present in large numbers and were more abundant in pool S than in pool NS. The phylogenetic diversity of these OTUs revealed that several Hymenobacter taxa were represented (see Fig. S3A in the supplemental material). To further assess the overrepresentation of Hymenobacter in “*Ca*. Phytoplasma solani”-negative declining lavenders, a quantitative real-time PCR assay was set up. Hymenobacter sp. genetic diversity is documented at 16S rRNA but also at the *gyrB* gene level. Degenerate primers designed from all Hymenobacter *gyrB* sequences were used to amplify *gyrB* from pools S and NS. Cloned sequences confirmed the diversity of Hymenobacter populations in both pools (Fig. S3B). Degenerate real-time PCR primers were designed with degeneration at variable positions. Three plasmids with inserts representative of *gyrB* diversity were selected to be used in equal mixtures as a quantitative standard to optimize a quantitative real-time PCR assay (see Fig. S4A in the supplemental material). When testing individually the DNA extracts of pools S and NS, the number of *Hymenobacter gyrB* copies was found to be not significantly different between “*Ca*. Phytoplasma solani”-negative symptomatic and nonsymptomatic lavenders (Fig. S4B). In contrast, the number of *Hymenobacter gyrB* copies was slightly lower in DNA extracts from “*Ca*. Phytoplasma solani”-positive declining lavenders than in nonsymptomatic lavenders collected at the same time from the same fields.

## DISCUSSION

### LD results from chronic infection by “*Ca*. Phytoplasma solani” lavender-specific strains.

“*Ca*. Phytoplasma solani” is the agent of many crop diseases in the Euro-Mediterranean basin and is usually transmitted by planthoppers of the family Cixiidae from perennial weeds (7–10, 15, 20, 21). These polyphagous planthoppers are univoltine in Europe and overwinter as larvae on the root systems of their wild plant hosts, from which they usually acquired the phytoplasma. In summer, infectious adults fly away in search of a new plant host and transmit the phytoplasma to a large number of cultivated and wild plants. In some specific crop rotations, infected larvae can develop on an intermediate plant host, as is the case for Reptalus panzeri, which completes its development on Johnsongrass (Sorghum halepense) or panzer wheat (Triticum aestivum) roots, before adults move to and inoculate maize fields (22). The main epidemic cycle of LD seems restricted to lavenders, as the “*Ca*. Phytoplasma solani” strains detected in lavenders are genetically different from the strains detected in surrounding bindweeds. At the beginning of the survey, some strains of *secY* genotypes S1, S4, and S6 could be detected in lavender fields, but genotypes S17, S16, and S14 became largely predominant over seasons. The multilocus sequence typing did not evidence any genetic difference between different isolates of genotypes S17, S16, and S14. These 3 genotypes have not been detected so far in any other crop in Europe. Genotype S14 was already present in the 1970s in fields of L. intermedia cv. Abrial, as it could be transmitted to C. roseus periwinkle using dodder Cuscuta subinclusa (3) and since then has been maintained in this host by grafting. According to the genotyping of the variable marker STAMP gene, which is widely used in Europe to trace “*Ca*. Phytoplasma solani” epidemics (23, 24), the S14 strain was introduced from Southeastern Europe, likely from Bulgaria, where lavender is also cultivated. The two other prevalent strains with genotypes S17 and S16 have probably evolved from local strains, as suggested by the clustering of their STAMP gene sequence with French “*Ca*. Phytoplasma solani” strains. Because of the current predominance of genotype S17, genotypes S14 and S16 may be remains of an old epidemic crisis that persists in the lavender production due to vegetative propagation. According to models for invasion and persistence of pathogens, persistence also requires that the host population provide a sufficient supply of susceptible individuals to maintain both the host and the pathogen populations, so that there is coexistence of the pathogen and host over long periods (25). Resistance to a specific strain of the pathogen can promote its elimination; however, such resistance to “*Ca*. Phytoplasma solani” could not be observed among lavender cultivars. As it is often the case for other plant yellows, multiple phloem-limited bacteria can induce the same disease (5, 26, 27). We therefore investigated the presence of other phloem-limited bacteria by universal phytoplasma detection and 16S rRNA deep sequencing in declining “*Ca*. Phytoplasma solani”-negative lavenders. Except for a few cases of “*Ca*. Phytoplasma trifolii,” no other bacterium was associated with LD. This phytoplasma was present in lavender fields as transmission assays with the leafhopper Cechenotettix martini in the early 1970s resulted in peculiar proliferation symptoms known to be induced by this phytoplasma present in the Mediterranean area (19, 28, 29). H. obsoletus has long been suspected to be the vector of lavender decline due to its abundance on declining lavender fields and its ability to fulfill its life cycle on lavender (12, 29). Transmission of LD by H. obsoletus under field conditions was obtained in the 1990s, but correlation with the detection of “*Ca*. Phytoplasma solani” was not fully conclusive, certainly due to the lack of efficiency of molecular detection tests available at that time (E. Boudon-Padieu, personal communication). As we were able to transmit the major genotype S17 to C. roseus periwinkle using infectious H. obsoletus collected in lavender fields, and because H. obsoletus insects collected on lavenders were also infected with S17, S16, and S14 “*Ca*. Phytoplasma solani” strains, this planthopper is hypothesized to be the main vector of LD. According to microsatellite analyses, H. obsoletus populations collected in French lavenders differ from populations living on C. arvensis and U. dioica in Europe (30). H. obsoletus can locally adapt to new plant hosts, developing into plant-specialized ecotypes that can be morphologically identical but that have limited exchange with other populations living on different plants (31, 32). It was recently shown that H. obsoletus populations developing on clary sage, a crop of the lavender plant family, had limited exchange with populations living on surrounding lavenders (33). According to these authors, clary sage appeared to be a poor host for “*Ca*. Phytoplasma solani” and did not host the lavender-specific strains of the phytoplasma, although they were highly abundant in the surrounding lavender fields. In conclusion, both “*Ca*. Phytoplasma solani” and H. obsoletus populations present in lavender fields appear to have undergone adaptation to lavender, and this could explain the lavender-specific epidemic cycle responsible for the fast propagation of LD.

Our results also show that high disease severity correlates with high frequency of “*Ca*. Phytoplasma solani” detection and that the phytoplasma population fluctuates over time, usually reaching a maximum at autumn. Except for the effect of warm summer temperature on phytoplasma multiplication late in the season, the reasons for phytoplasma population fluctuation in lavender are poorly understood. The temporal evolution of the symptoms is characterized by the regular increase of dying canopy sectors that certainly constitutes for lavender a means to control the phytoplasma propagation. It is likely that competition between lavender defenses and multiplication of the phytoplasma results in an incomplete elimination of the pathogen, which recolonizes the rest of the plant.

### Consequence for LD control and sustainability of lavender production.

The first impact of LD is yield loss resulting from lavender death. The quality loss in essential oil is still poorly evaluated. It is known, for instance, that “*Ca*. Phytoplasma solani” not only causes low harvest but also affects the quality of wine made from susceptible cultivars of Vitis vinifera (34). Sustainability of lavender production will rely on the prophylactic control of LD but will also have to take into account climate warming, which may influence the fitness and geographic spread of both phytoplasma and insect populations. Little is known about the impact of temperature on “*Ca*. Phytoplasma solani,” especially on the multiplication of the lavender-specific strain of the phytoplasma. An increase of two degrees has been shown to enhance the multiplication of the flavescence dorée phytoplasma, with drastic impact on the development of the experimentally infected plants (35). The transmission of the “*Ca*. Phytoplasma solani” strain of genotype S17 will help to determine its optimal temperature for growth and hence concentrate prophylactic control on geographical areas at higher risk for phytoplasma propagation. The risk reduction option based on the planting of resistant cultivars is not feasible in the short term, as no resistance to “*Ca*. Phytoplasma solani” could be observed among lavender cultivars and as cultivars thought to be tolerant reach phytoplasma populations equivalent to those of susceptible cultivars (36). So, reduction of LD epidemics will rely on the production of phytoplasma-free lavender for planting pathogen-free fields, the elimination of highly infected fields, and the deployment of new cultural practices to reduce insect vector populations. Elimination of reservoir weed plants is known to be effective against the spread of bois noir disease of grapevine (37, 38). As genotypes S1 and S4, which are usually associated with bindweeds, can be detected the first years following plantation, weeding should reduce part of the initial LD inoculum. However, as we showed that the lavender-specific genotypes S17, S14, and S16 take over the following years, the elimination of reservoir weed plants may have a limited impact in the long term to reduce LD epidemics. Using physical barriers aimed to protect newly planted fields from H. obsoletus colonization is a potential risk reduction option. Lavender plants grown for flowers are now grown under insect-proof tunnels, as the income of this production is high enough to cover the additional cost of protecting infrastructure. For essential oil and lavender honey production, lavender is cultivated in open fields. The production of “*Ca*. Phytoplasma solani”-free certificated planting material is currently promoted, but young fields must be protected from the installation of H. obsoletus and the introduction of the phytoplasma inoculum. As no insecticide can be used in such production, alternative methods such as intercropping and kaolin sprays are currently being tested. H. obsoletus is a xerothermic insect, and females prefer warm and nude soil in which to lay eggs; covering the soil is therefore one of the most promising cultural practices. The combination of such new cultural practices with certification programs and increased collective eradication should improve LD management.

## MATERIALS AND METHODS

### Disease survey, plant and insect collection, and transmission assay.

Twenty lavender fields of different cultivars representative of lavender production and located in the four French departments where lavender is cultivated were selected for the study (Table 4). As some of the lavender fields surveyed had been pulled out at the end of 2008 due to the high incidence of decline, 7 new fields were introduced in the survey in 2009. The incidence of LD was measured by counting declining plants among 1,000 plants in May and June 2008, 2009, and 2010 as well as in October and November 2008 and 2009. Each diseased plant was scored using a severity index. Healthy plants were scored 0, plants with low vigor were noted 1, yellowing plants were noted 2, and plants presenting up to 25%, 26% to 50%, 51 to 75%, and 76 to 99% of their canopy dried were scored 3, 4, 5, and 6, respectively. Dead plants were scored 7. In spring and autumn 2008 and 2009, 15 declining plants as well as 4 healthy controls were randomly sampled in each field by collecting 3 to 5 shoots per plant. In 2008 and 2009, the plants sampled were different, whereas in 2010, a resampling strategy was carried out to evaluate the stability of noninfection status of the lavenders and evaluate the rate of the epidemic spread of “*Ca*. Phytoplasma solani.” In detail, in spring 2010 the samples corresponded to declining lavenders that previously tested negative for “*Ca*. Phytoplasma solani” in 2008, whereas in autumn 2010 the samples corresponded to declining lavenders that previously tested negative for “*Ca*. Phytoplasma solani” in 2009. A healthy control sample corresponding to a greenhouse-grown seedling of lavender complemented each field sample set.

**TABLE 4:** Description of lavender fields surveyed

Field code	Location	Species (cultivar)	Yr planted	Altitude (m)
RM	Saint Trinit (Vaucluse)	L. angustifolia (Maillette)	2006	912
PP^*c*^	Champlong, Sault (Vaucluse)	L. angustifolia (Fine, Population)	2007	1,005
BL	Les Laurences (Vaucluse)	L. angustifolia (Rapido)	2005	973
CC^*b*^	Saint Christol (Vaucluse)	L. angustifolia (C15-50)	2004	848
RJ	Férrassières (Drôme)	L. angustifolia (Rapido)	2005	1,018
MT	Saint-Auban-sur-l'Ouvèze (Drôme)	L. angustifolia (Maillette)	2006	627
MTF^*b*^	Die (Drôme)	L. angustifolia (Matherone)	2004	385
RT	Die (Drôme)	L. angustifolia (Rapido)	2005	462
BM^*c*^	Mévouillon (Drôme)	L. angustifolia (Bleue)	2007	875
PPi	L'Epine (Hautes-Alpes)	L. angustifolia (Fine, Population)	2004	1,067
AA	Chamaloc (Drôme)	L. intermedia (Abrial)	2006	593
AS	Sault (Vaucluse)	L. intermedia^*a*^ (Abrial)	2004	706
GS	Saint-Auban-sur-l'Ouvèze (Drôme)	L. intermedia (Grosso)	2004	600
SS	Saint-Auban-sur-l'Ouvèze (Drôme)	L. intermedia (Sumian)	2006	600
RS	Revest du Bion (Alpes-de-Haute-Provence)	L. intermedia (Super)	2005	933
SB	Entrevennes (Alpes-de-Haute-Provence)	L. intermedia (Super)	2004	643
GD	Valensole (Alpes-de-Haute-Provence)	L. intermedia (Grosso)	2004	600
GC	Puimoisson (Alpes-de-Haute-Provence)	L. intermedia (Grosso)	2007	736
GM	La Rochegiron (Alpes-de-Haute-Provence)	L. intermedia (Grosso)	2006	758
SP	Banon (Vaucluse)	L. intermedia (Super)	2006	705
PF	Sault (Vaucluse)	L. angustifolia (Fine, Population)	2005	1,005
BJ	Mévouillon (Drôme)	L. angustifolia (Bleue, Population)	2008	841
SL	Banon (Vaucluse)	L. intermedia (Super)	2008	758
AA2	Chamaloc (Drôme)	L. intermedia (Abrial)	2008	551
AF	Sault (Vaucluse)	L. intermedia (Abrial)	2008	744
GF	Mévouillon (Drôme)	L. intermedia (Grosso)	2009	875
GG	Grignan (Drôme)	L. intermedia (Grosso)	2008	200

a L. intermedia plants are *L. angustifolia × L. latifolia* hybrids.

b Field pooled out in early 2010.

c Field pooled out in autumn 2008.

A total of 3,056 lavender samples were collected in 22 nurseries over a 3-year period (2008 to 2010). Eleven nurseries under insect-proof tunnels contained certified propagated material that originated from *in vitro*-regenerated grandmother stocks that tested negative for “*Ca*. Phytoplasma solani.” The remaining 11 nurseries grew plants in open fields either from certified or from noncertified mother plants. Plants were sampled upon survey for pale leaves or lack of vigor or by random sampling when no symptoms were observed.

Nymphs of H. obsoletus were observed by pulling out 10 plants and counting the larvae on the root system. Adults of H. obsoletus were captured by 15-min mechanical aspiration with D-Vac on 20 lavender plants. Transmission assays to C. roseus periwinkle seedlings were performed by delivering 20 adults per plant, and assays lasted until all insects died. Plants were kept at 20 to 25°C with a 16-h photoperiod until the appearance of symptoms. Symptomatic “*Ca*. Phytoplasma solani” isolate Champlong (genotype S17) as well as the “*Ca*. Phytoplasma solani” reference strain Dep transmitted from L. intermedia cv. Abrial through C. subinclusa (genotype S14) (3) was maintained in periwinkle by top grafting new C. roseus seedlings.

### Nucleic acid extraction.

Lavandula samples consisted of three to five herbaceous ramifications with decline symptoms collected from one plant. Leaves were removed with a razor blade, and the remaining stems were cut into small pieces and pooled. Only 0.5 g of stem was used for nucleic acid extraction, and the rest was kept frozen at −20°C for further use. For other plants, leaf midribs and petiole were used. Plant tissues were ground and homogenized with a hydraulic press before proceeding to total nucleic acid extraction using the cethyl-trimethyl-ammonium bromide (CTAB) protocol as described by Daire and colleagues (39). The final DNA pellet was dissolved in 50 μl of Tris-EDTA (TE) buffer (10 mM Tris, 1 mM EDTA, pH 7.6).

### Quantitative PCR detection of “*Ca*. Phytoplasma solani” in plants and insects.

The “*Ca*. Phytoplasma solani” detection assay was adapted from the detection of “*Ca*. Phytoplasma solani” in grapevine (14). The “*Ca*. Phytoplasma solani”-specific TaqMan MGB probe (Stol) was 5′ labeled with 6-carboxy-2,4,4,5,7,7-hexachlorofluorescein (HEX) reporter dye. For the plant endogenous control, two primers for the plant DNA amplification and a plant TaqMan probe were designed within the cytochrome oxidase subunit I gene (COI) on the basis of alignment of various COI plant sequences. The primers were f-Cox (5′-CGTCGCATTCCAGATTATCCA-3′) and r-Cox (5′-CAACTACGGATATATAAGAGCCAAAACTG-3′). The COI TaqMan MGB probe (5′-TGCTTACGCTGGATGGAATGCCCT-3′) was 5′ labeled with 6-carboxyfluorescein (FAM) as the reporter dye. Reactions were performed in a final volume of 20 μl containing 10 μl of QuantiTect multiplex PCR buffer (Qiagen, Hilden, Germany), 0.2 μM each primer and COI probe, 0.4 μM Stol probe, and 1 μl of purified nucleic acid. Assays were performed on an MX 3005 apparatus (Stratagene, La Jolla, CA, USA). The cycling conditions were as follows: 15 min at 95°C for Hot Start *Taq* DNA polymerase activation, followed by 50 cycles of 94°C for 60 s and 59°C for 90 s. The estimation of the cycle threshold (*C_T_*) was calculated with MXPro v4.10 software (Stratagene, La Jolla, CA, USA). In each PCR plaque, three healthy lavenders and three “*Ca*. Phytoplasma solani”-positive lavenders were used as controls. Samples with *C_T_* of <40 were considered positive.

For a time course quantitative survey of “*Ca*. Phytoplasma solani” infection, a quantitative reference was added to the assay. It consisted of a plasmid containing the gene target as the insert. Amplification of a 976-bp fragment of the “*Ca*. Phytoplasma solani” strain PO *map* gene was achieved using primers adkF2 (5′-GTTGGTCGCAGAATTTGTCC-3′) and if1R2 (5′-CCAGAAACATAAGCGGTAATCGT-3′) for 35 cycles (94°C for 40 s, 55°C for 40 s, 72°C for 40 s). The *map* PCR product was cloned in pGEM-T easy plasmid (Promega, Charbonnières-les-Bains, France) according to the manufacturer's instructions. Results were analyzed using MxPro QPCR software (Agilent, Les Ulis, France). The number of phytoplasmas was calculated for 200 ng of lavender total nucleic acids.

### Universal phytoplasma detection.

Detection of phytoplasma infection was performed on 1 μl of nucleic acid extract by 16S rRNA nested PCR with the universal primers for phytoplasmas R16mF2/R16mR1 followed by R16F2n/R16R2 according to the PCR conditions described in the original publication (40), except that dilution of the first PCR was omitted. The sequence of the PCR product was compared to that of reference 16S rRNA to identify the “*Ca*. Phytoplasma” species.

### Genotyping of “*Ca*. Phytoplasma solani” strains.

Nested-PCR amplification of “*Ca*. Phytoplasma solani” *secY* gene was performed as previously described using the POsecF1-POsecR1 primer pair for the first PCR and the POsecN2-POsecR3 primer pair for the nested-PCR step (41). Amplified products were directly sequenced on both strands on the MegaBACE capillary sequencer by Beckman Coulter Genomics (Takeley, UK). Raw chromatograms were assembled and edited using the Phred-Phrap-Consed package (42–44). “*Ca*. Phytoplasma solani” *secY* sequence accession numbers are indicated on Fig. S1 in the supplemental material [see also “Accession number(s)” below]. The “*Ca*. Phytoplasma solani” strain Champlong transmitted to C. roseus periwinkle and the LD strain Dep transmitted to C. roseus in 1970 were further characterized by nested-PCR amplification and sequencing of the housekeeping gene *tuf* and the two genes *vmp1* and STAMP gene, encoding variable surface proteins, according to previously published procedures (24, 41, 45, 46).

### Deep sequencing of V4V5-amplified 16S rRNA.

Pools of lavender total nucleic extracts from autumn 2009 consisted of mixtures of equal volumes of 22 extracts of “*Ca*. Phytoplasma solani”-free nonsymptomatic lavenders (pool NS) and of 30 extracts of “*Ca*. Phytoplasma solani”-free declining lavenders from the same fields (pool S). Pool NS contained 7 L. angustifolia plants (fields MT, MTF, RJ, CC) and 16 L. intermedia plants (fields GS, GD, SP, RS, SB, AA, AS), whereas pool S contained 12 L. angustifolia and 21 L. intermedia plants from the same fields. Total nucleic acid concentration was adjusted to 50 ng/μl in each pool and submitted to amplification of the V4V5 region of bacterial 16S rRNA (47) and Solexa sequencing performed on an Illumina MiSeq apparatus. After trimming for phred quality 30 and minimum length of 100 bases, reads were clustered into operational taxonomic units (OTUs) using CROP (clustering 16S rRNA for OTU prediction) at a 97% similarity threshold (48), and taxonomic assignments were performed by BLAST comparison against the GenBank database. The representation of OTUs was the number of reads of each pool constituting a given OTU. Phylogenetic analysis of Hymenobacter sp. OTUs was conducted as described below.

### Diversity analysis of Hymenobacter sp. *gyrB* sequences and quantification of hymenobacters.

Conserved regions within *gyrB* gene sequences were identified by aligning Hymenobacter species *gyrB* sequences. *gyrB* GenBank accession numbers are indicated on Fig. S2B in the supplemental material. Two sets of primers were designed in these conserved regions to conduct nested-PCR amplification carried out with New England BioLabs *Taq* DNA polymerase. Total nucleic acids of pool S and pool NS were submitted to a first amplification with distal degenerated primers HYBGyrB-F0B (5′-GTRGARGTRGCSYTSCARTACAA-3′) and HYBGyrB-R0B (5′-CGGTTRCGGCCYTGYTTGGC-3′) with PCR conditions of 3 min at 95°C and 35 cycles of 30 s at 95°C, 30 s at 60°C, and 30 s at 72°C. Nested-PCR amplification was performed using the degenerated primers HYBGYRB-FOR1 (5′ TACGTCAACAACATCAAYAC 3′) and HYBGYRB-R1 (5′ GAGCAGTCRGCCAGRTTRCC 3′) and the same PCR conditions to amplify PCR products of 408 bp. PCR products were ligated to pGEMt-easy (Promega) and cloned into Escherichia coli strain DH10B by electroporation according to standard procedures. Inserts of 10 recombinant plasmids corresponding to each pool were sequenced as described above.

A real-time PCR quantification of Hymenobacter genome copies was developed based on *gyrB* polyvalent amplification. Primers for real-time PCR were designed on the basis of the alignment of *gyrB* gene sequences from the Hymenobacter species indicated in Fig. S2B and 13 *gyrB* sequences cloned from lavender pools S and NS. Primers HYBGYRB-FOR1 (5′-TACGTCAACAACATCAAYAC-3′) and HYBGYRB-REV (5′-CCYTCVCGGAAGTCNTCDCC-3′) were designed to amplify a 149-bp-long fragment from all hymenobacter *gyrB* genes. Standard curves for the absolute quantification of hymenobacters were obtained by testing serial dilutions in DNA-free water of the standard plasmids from 1E8 to 1E3 copies tested in triplicates. The standard plasmids corresponded to the equimolar mixture of 3 plasmids with different inserts, as indicated in Fig. S2B. Samples corresponded to 25 ng of DNA from RNase A-treated nucleic acids from the 23 extracts from the pool NS and 30 extracts from pool S as well as 29 extracts from “*Ca*. Phytoplasma solani”-positive diseased lavender from the same fields sampled at the same collection date. Real-time PCR assays were conducted using LightCycler 480 SYBR green I Master (Roche, La Rochelle, France) and primers at 0.4 μM. Amplification was performed in 96-well plates (LightCycler 480 Multiwell Plate 96) on a 480 LightCycler real-time PCR detection system with the following cycles: 1 cycle at 95°C for 15 min and 40 cycles of 94°C for 15 s, 54°C for 30 s, and 68°C for 20 s. Data acquisition and analysis were handled by the LightCycler 480 software release 1.5.0. (Roche, La Rochelle, France), which automatically calculates the number of *gyrB* gene copies in each sample by comparison with the standard range. *gyrB* is known to occur as a single copy in bacterial genomes. Comparison of the data and statistical significance were determined using one-way analysis of variance (ANOVA) with the software R (Development Core Team, 2014) and the package R Commander (49).

### Phylogenetic analyses.

Multiple alignments of homologous sequences retrieved from GenBank were performed for “*Ca*. Phytoplasma solani” *secY* and STAMP gene sequences, Hymenobacter sp. 16S rRNA, and *gyrB* sequences using ClustalW (50). Phylogenetic analyses were conducted with MEGA6 using the maximum parsimony method and bootstrapping 250 times to estimate branching stability (51, 52).

### Accession number(s).

Genes for the antigenic membrane protein STAMP of “Candidatus Phytoplasma solani” have been deposited in the NCBI database under BioProject PRJEB25879. Genes for the preprotein translocase SecY have been deposited in the NCBI database under accession numbers FN813271 to FN813290 and HF969329 to HF969334.

## Supplementary Material

Supplemental file 1
