# Supplementary material for: Lavender Decline in France Is Associated with Chronic Infection by Lavender-Specific Strains of “Candidatus Phytoplasma solani”
Source: Appl Environ Microbiol. 2018 Nov 30;84(24):e01507-18. doi: 10.1128/AEM.01507-18 (PMC6275342; doi:10.1128/AEM.01507-18)
Supplement: Supplemental file 1 [file zam023188881s1.pdf]

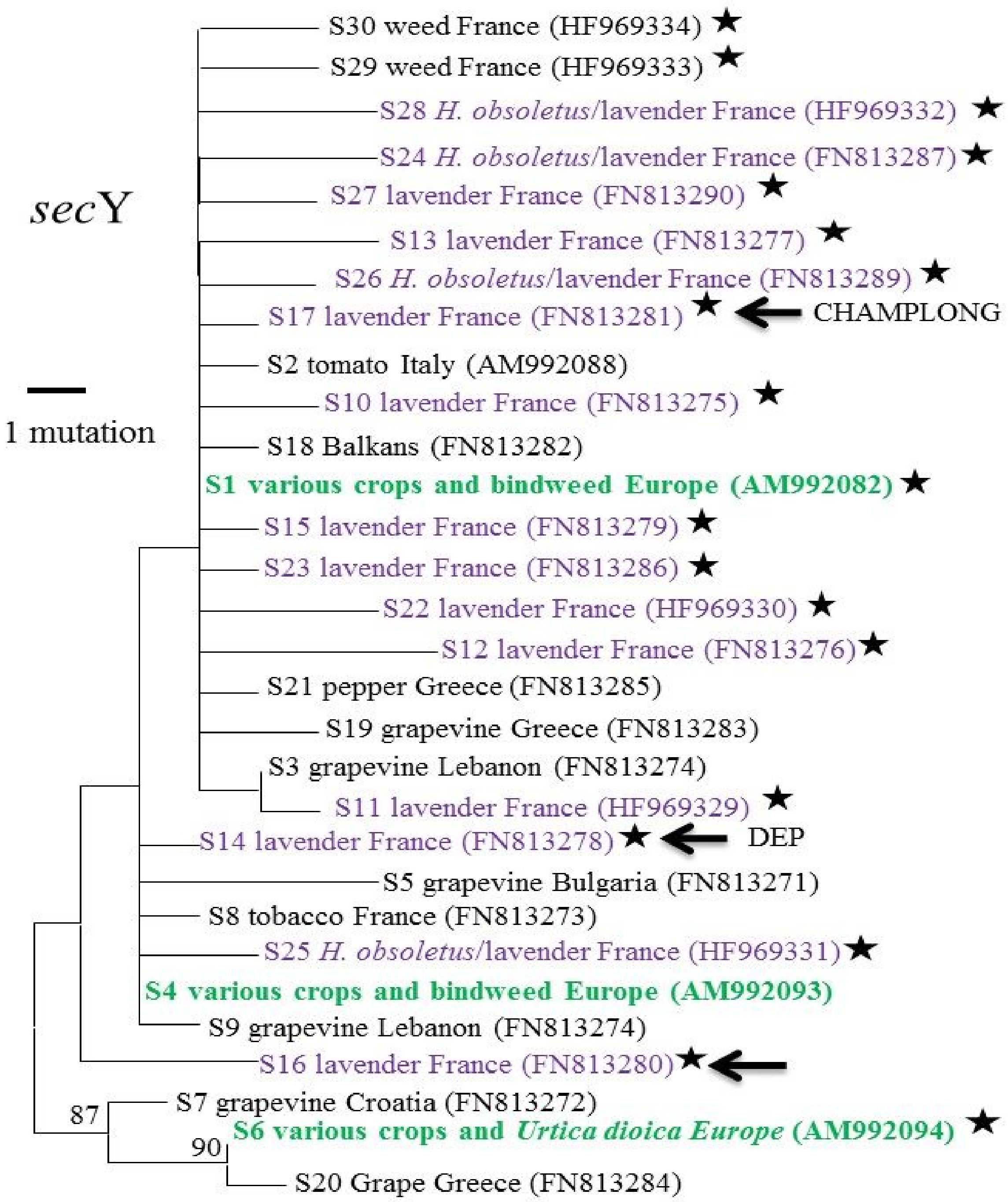

FIG S1 Genetic characterization of "*Candidatus* Phytoplasma solani" strains detected in lavender fields. (A) Maximum Parsimony analysis of "*Candidatus* Phytoplasma solani" *sec*Y genes from declining lavenders and *Hyalesthes obsoletus* collected in declining lavender fields. One of the 10 parsimonious trees is shown. The percentage of replicate trees in which the associated taxa clustered together in the bootstrap test (250 replicates) are shown next to the branches. All positions containing gaps and missing data were eliminated. There were a total of 727 positions in the final dataset. Sequences colored in green correspond to the 3 most abundant genotypes found in Europe and the 17 sequences detected in lavender fields are highlighted in with stars and those detected in lavender are colored in purple. '*Ca.* P. solani' reference strains DEP and CHAMPLONG originating from lavender and maintained in Madagascar periwinkle are indicated by arrows. Accession numbers are indicated between brackets

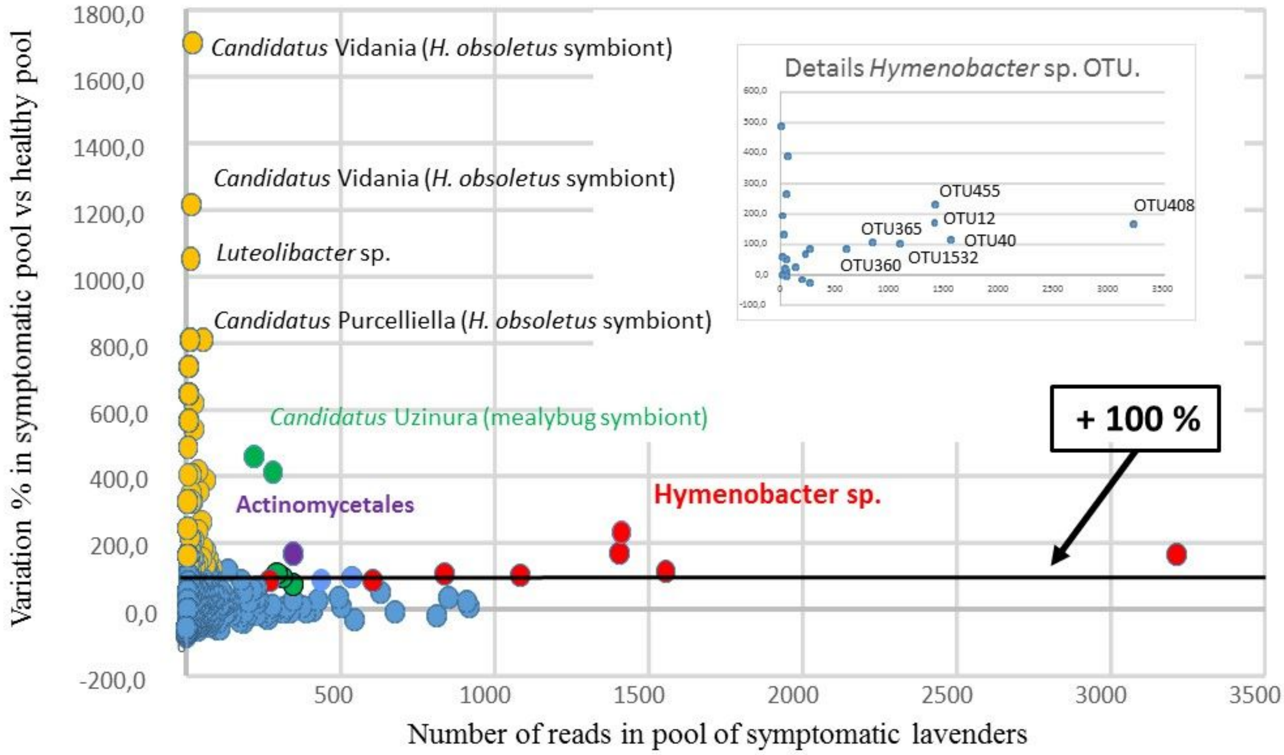

FIG S2 Relative variation of bacterial OTUs prevalence in phytoplasma-free declining lavenders versus healthy lavenders.

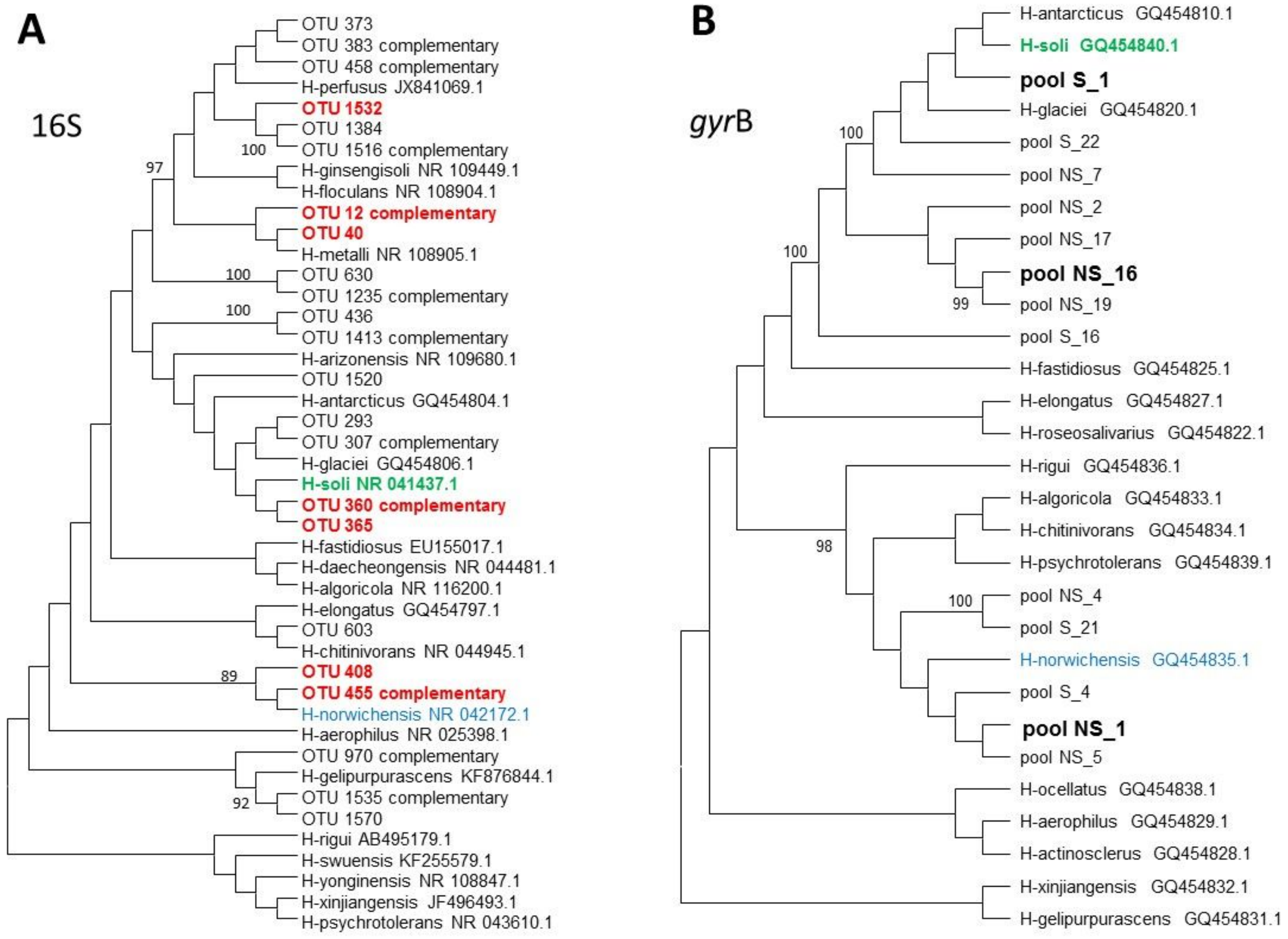

FIG S3 Maximum parsimony phylogenetic analyses using of V4V5 regions of *Hymenobacter* sp. OTU (16S rRNA) and *gyr*B sequences present in lavenders negative for '*Ca.* P. solani'. Consensus trees builded from: **A:** Phylogenetic analysis of bacterial 16S OTU homologous to genus Hymenobacter 16S rRNA sequences detected in declining and healthy lavenders with *Hymenobacter* sp. 16S rRNA sequences available in Genbank. OTU sequences labelled in red correspond to the most represented OTUs (see Fig 6). **B:** Phylogenetic analysis of *gyr*B sequences cloned from *gyr*B amplicons amplified from pools of nucleic acids. GyrB clones from declining lavenders are noted S and from healthy lavenders are noted NS. The three sequences in bold characters correspond to the insert of the three plasmid clones that served in equal mixture as quantitative standards (see Fig. S4). The percentage of replicate trees in which the associated taxa clustered together in the bootstrap test (250 replicates) are shown next to the branches.

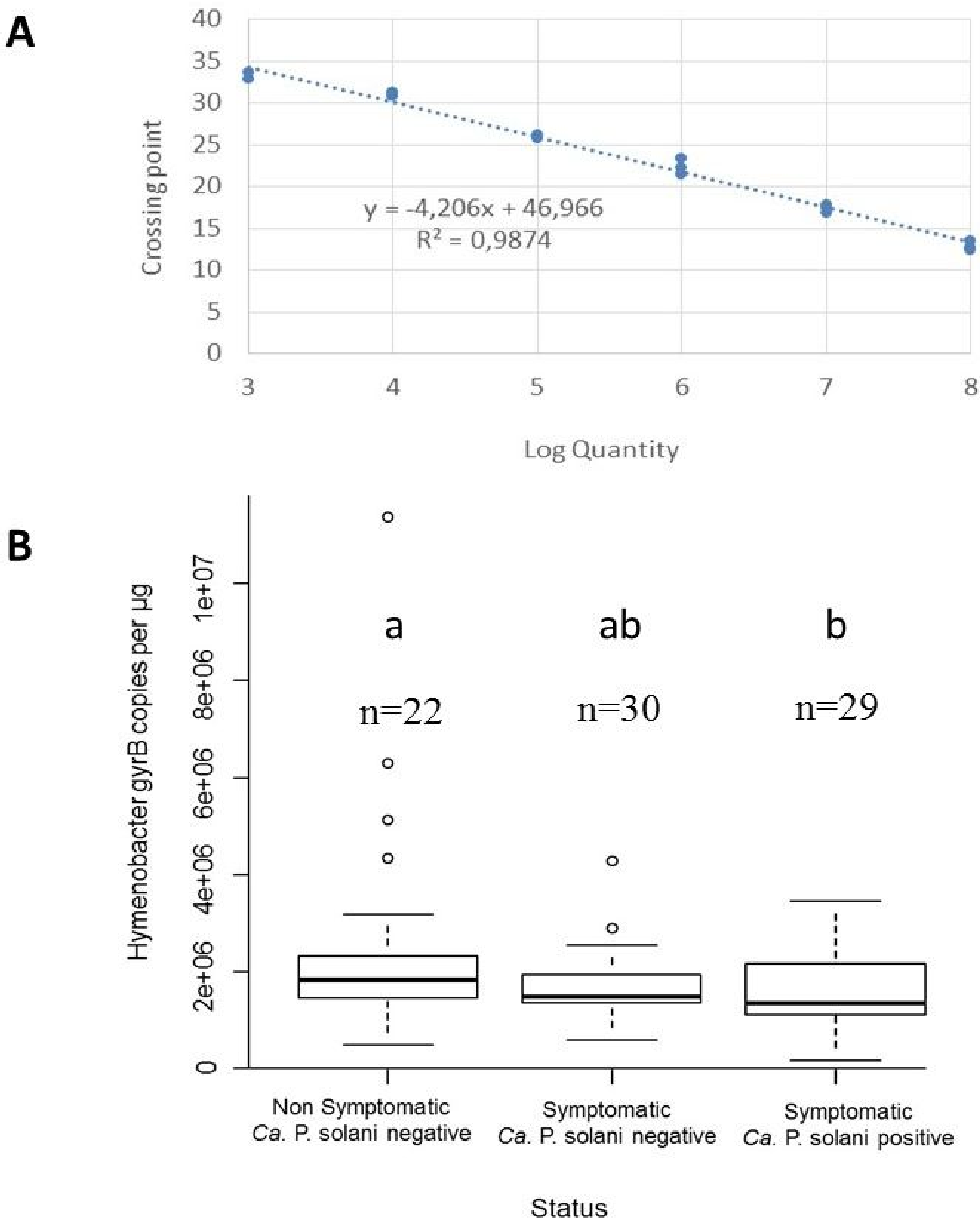

FIG S4: Quantification of *Hymenobacter* in lavenders. **A:** Calibration curve obtained after 10 fold dilution series of equal mixture of the three plasmids S_1, NS_1 and NS_16 representative of representative of *gyr*B genetic diversity in French lavenders. **B.** Detection of gyrB copy numbers in DNA of healthy or declining lavenders negative for "*Ca.* P. solani" detection and in DNA of "*Ca.* P. solani"-positive declining lavenders. Letters **a** and **b** indicate groups statistically different in ANOVA. N is the number of individual lavender DNA extracts tested.

Supplementary table S1: Prevalence of "*Ca.* P. solani" in *L. angustifolia* and *L. intermedia* in nurseries during the 2008-2010 period.

Type of nursary	Number of nurseries surveyed	"*Ca.* P. solani" PCR positive/total tested (% of positives)	Number of infected nurseries
Certified grand-mother stocks grown under insect-proof tunnels	1	0/597	0
Certified mother stocks and planting material grown under insect-proof tunnels	10	4/574	1
Certified *L. intermedia* Grosso grown in open fields	6	11/1269	1
Non certified planting material grown in open fields	5	59/616	5

Supplementary table S2: Incidence of "*Ca.* P. solani" positive plants in declining plants at spring and autumn 2010 among plants previously tested negative

Season of testing – season of previous negative test	*L. intermedia* [5] PCR positive/tested (% of positive)	*L. angustifolia*[6] PCR positive/tested (% of positive)
Spring 2010 – autumn 2008[1]<br>Spring 2010 – spring 2008[2]	11/96 (11.5%)<br>17/91 (18.7%)	9/63 (14.3%)<br>22/50 (44%)
Autumn 2010 – autumn 2009[3]<br>Autumn 2010 – spring 2009[4]	18/58 (31%)<br>28/78 (35.9%)	11/30 (36.7%)<br>16/34 (47%)

[1] plants exposed during summer 2009 after negative test, [2] plants exposed during summers 2008 and 2009 after negative test, [3] plants exposed during summer 2010 after negative test, [4] plants exposed during summers 2009 and 2010 after negative test

[5] collected in the 10 fields GS, GD, SB, GC, SP, AS, GM, AA, SS and RS

[6] collected in the 6 fields RM, BL, RJ, RT, MTF and PPi

Supplementary table S3: Distribution of "*Ca.* P. solani" *sec*Y genotypes in lavender fields

	Number of plant infected by the secY genotype														
Field	S1	S4	S6	S10	S11	S12	S13	S14	S15	S16	S17	S22	S23	S27	S28
GS	2		1					1		2	1	1			
AS	2			1	1	1		2		1	14				
CC							1	3	1		5		1		
RJ		1	1					2		7	6				
RS											1				
MTF	1									1	1				
RT	1							8		4	8			1	
BL								4		1	14				
RM								1		2	18				
GC								1			8				
SP		1									4				
GM											6				
SS										6	7				
PPi		2						2		1	4				
PP								1		1	8				
AA								12		2	5				1
MTF								2		4	1				
SB								3			10				
SL											6				
BJ	1							2		6	6				
PF								1			6				
CS										1	5				

Supplementary table S4: multilocus genotypes of "*Ca.* P. solani" isolates detected in lavenders and reference strains propagated in *C. roseus* periwinkle.

Isolates code or strain name	host	*tuf*	*sec*Y	*vmp*1	*stamp*
L2262	*Lavandula intermedia* cv Super	tuf-b1	S17	V4	ST20
L2275	*L. angustifolia* cv Fine	tuf-b1	S17	V4	ST20
L3981	*L. angustifolia* population	tuf-b1	S17	V4	ST20
L2664	*L. intermedia* cv Abrial	tuf-b1	S17	V4	ST20
CHAMPLONG	*Catharanthus roseus*	tuf-b1	S17	V4	ST20
L3991	*L. angustifolia* population	tuf-b2	S14	V1	ST10
L2674	*L. intermedia* cv Abrial	tuf-b2	S14	V1	ST10
DEP	*C. roseus*	tuf-b2	S14	V1	ST10
L2667	*L. intermedia* cv Abrial	tuf-b1	S16	V4	ST5
L4222	*L. intermedia* cv Sumian	tuf-b1	S16	V4	ST5

Supplementary table S5: results of "*Ca.* P. solani" real-time PCR and *sec*Y genotypes detected on weeds collected in the surroundings of lavenders fields

Year	Fields	positive weeds/ total tested	*sec*Y genotype detected (Plant) [1]
2008	RS, SP, AS, RJ, AA, PPi, MTF, RT, GM, SS, GS	17/117	S29 (1 unidentified)<br>S30 (1 unidentified)<br>S4 (1 *Daucus carota* wild carrot)<br>2 S1 (1 *Rubus fruticosus* blackberry, 1 *Convolvulus siculus*)<br>S17 (1 *Convolvulus arvensis* field bindweed)
2009	BJ, RJ, SP, SL, PPi, GS, SS, AA, RT, PF, AF, GF, GG, AS	9/39	2 S1 (2 *C. arvensis* field bindweeds)<br>3 S4 (2 *C. arvensis* field bindweeds, 1 wild lavender)
2010	RM, CS, BL, GG, PPi, GM, SL, BJ, SS, RJ, AF, GF, AA, RS, PF	16/89	2 S1 (2 *C. arvensis* field bindweeds)<br>S4 (1 *C. arvensis* field bindweed)

[1]28 samples gave a real-time PCR Ct value higher than 35 and did not give amplification with the *sec*Y nested-PCR assay

Supplementary table S6: Sequences used for *stamp* phylogenetic analyses

Country	Isolate	Biological origins	Accession number	Reference
France	L2667-S16	*Lavandula intermedia* cv. Abrial	PRJEB25879	Present publication
France	L4222-S16	*L. intermedia* cv. Sumian	PRJEB25879	Present publication
France	L2674-S14	*L. intermedia* cv. Abrial	PRJEB25879	Present publication
France	L3991-S14	*L. angustifolia* Fine population	PRJEB25879	Present publication
France	L4009-S14	*L; intermedia* cv. Abrial	PRJEB25879	Present publication
France	L4300-S14	*L. angustifolia* Fine population	PRJEB25879	Present publication
France	DEP-S14	*Catharanthus roseus* inoculated from *L. intermedia* cv. Abrial by *Cuscuta campestris*	PRJEB25879	(1)
France	L2262-S17	*L. intermedia* cv. Super	PRJEB25879	Present publication
France	CHAMPLONG-S17	C. roseus inoculated by *Hyalesthes obsoletus* collected on *Lavandula angustifolia* Fine population	PRJEB25879	Present publication
France	L2275-S17	*L. angustifolia* Fine population	PRJEB25879	Present publication
France	L3981-S17	*L. angustifolia* Bleue population	PRJEB25879	Present publication
Germany	19-25	*C. campestris inoculated C. roseus* from *V. vinifera*	FN813267	(2)
Bulgaria	BG4560	*V. vinifera*	FN813252	(2)
Bulgaria	BG-2009-5899	*V. vinifera*	Identical KC703019	Foissac and Avramov, unpublished
Serbia	STOL	*C. campestris* inoculated *C. roseus* from *Capsicum annuum*	FN813261	(2)
Germany, Switzerland, France	HO-Ud-S1	*Urtica dioica*	JQ977713	(3)
Slovenia, Croatia, Italy	HO-Ud-S2	*H. obsoletus* collected on *U. dioica*	JQ977714	(3)
Slovenia, Croatia, Italy	HO-Ud-S3	*H. obsoletus* collected on *U. dioica*	JQ977715	(3)
Italy	HO-Ud-S4	*U. dioica*	JQ977716	(3)
Italy	HO-Ud-S5	*H. obsoletus* collected on *U. dioica*	JQ977717	(3)
Italy	HO-Ud-S6	*H. obsoletus* collected on *U. dioica*	JQ977718	(3)
Slovenia	HO-Ud-S7	*H. obsoletus* collected on *U. dioica*	JQ977719	(3)
Germany	LA6_I_C	*H. obsoletus* collected on *Convolvulus arvensis*	JQ977720	(3)
Serbia	Rpm35	*Reptalus panzeri* collected on *Zea mais*	KC703015	(4)
Serbia	Vv12_Kn6	*V. vinifera* cv. Zweigelt	KC703017	(4)
Serbia	Rqg50	*R. quinquecostatus* collected on *V. vinifera*	KC703019	(4)
Austria	CrHo12_650-St_At1	*H. obsoletus* inoculated *C. roseus*	KJ469716	(5)
Austria	Vv12_274-St_At2	*V. vinifera*	KJ469717	(5)

Austria	Ho13_1006-St_At3	*H. obsoletus*	KJ469718	(5)
Austria	CrHo13_1183 St_At4	*H. obsoletus* inoculated *C. roseus*	KJ469719	(5)
Austria	Ho13_838 St_At5	*H. obsoletus*	KJ469720	(5)
Austria	CrHo12 St_At6_601	*H.obsoletus* inoculated *C. roseus*	KJ469721	(5)
Austria	CrAr12_722_2 St_At7	*V. vinifera*, *H. obsoletus* inoculated *C. roseus, R. panzeri*	KJ469722	(5)
Austria	Vv12_751 St_At8	*V. vinifera*	KJ469723	(5)
Austria	Vv12_Kn6 St_At9	*V. vinifera*	KJ469724	(5)
Bosnia and Herzegovina	3-11	*V. vinifera*	KP739850	(5)
Bosnia and Herzegovina	30-09	*V. vinifera*	KP739851	(5)
Bosnia and Herzegovina	G21-13	*V. vinifera*	KP739856	(5)
Italy	Vercer-Marche	*V. vinifera*	KJ145375	(6)
Italy	P49-Marche	*V. vinifera*	KJ145376	(6)
Italy	GA4-Abruzzi	*V. vinifera*	KJ145377	(6)
Italy	B1-Campania	*V. vinifera*	KJ145378	(6)
Italy	P46-Marche	*V. vinifera*	KJ145379	(6)
Italy	A16-Abruzzi	*V. vinifera*	KJ145380	(6)
Italy	TE7-Abruzzi	*V. vinifera*	KJ145381	(6)
Italy	CA21-Marche	*V. vinifera*	KJ145382	(6)
Italy	AQ1-Abruzzi	*V. vinifera*	KJ145383	(6)
Italy	DXSAIN-Marche	*V. vinifera*	KJ145384	(6)
Italy	CIL-Marche	*V. vinifera*	KJ145385	(6)
Italy	TE17-Abruzzi	*V. vinifera*	KJ145386	(6)
Italy	A25- Abruzzi	*V. vinifera*	KJ145387	(6)
Italy	AQ29- Abruzzi	*V. vinifera*	KJ145388	(6)
Georgia	Carv1	*V. vinifera*	KT184879	(7)
Georgia	Carv2	*V. vinifera*	KT184880	(7)
Georgia	Char7	*V. vinifera*	KT184881	(7)
Georgia	Char8	*V. vinifera*	KT184882	(7)
Georgia	Amla77	*V. vinifera*	KT184883	(7)
Georgia	Sabu84	*V. vinifera*	KT184884	(7)
Georgia	Tsol89	*V. vinifera*	KT184885	(7)
Azerbaijan	AZ_GR05-2015	*V. vinifera*	LT899730-	(8)
Azerbaijan	AZ_GR21-14	*V. vinifera*	LT899734-	(8)
Azerbaijan	AZ_GR23-14	*V. vinifera*	LT899735	(8)
Azerbaijan	AZ_GR24-14	*V. vinifera*	LT899736	(8)
Azerbaijan	AZ13-H05	*H. obsoletus* collected on *C. arvensis*	LT899738-	(8)
Azerbaijan	AZ13-H40	*H. obsoletus* collected on *C. arvensis*	LT899749	(8)
Azerbaijan	AZ2007_AUB3	*Solanum melongena*	LT899753-	(8)
Azerbaijan	AZ2007_PV1	*C. annuum*	LT899754	(8)
Azerbaijan	AZ2008_TO41	*S. lycopersicum*	LT899756	(8)

References

1. Boudon-Padieu E, Cousin MT. 1999. Yellow decline of *Lavandula hybrida* Rev and *L. vera* DC. International Journal of Tropical Plant Diseases 17:1-34.
2. Fabre A, Danet J-L, Foissac X. 2011. The stolbur phytoplasma antigenic membrane protein gene stamp is submitted to diversifying positive selection. Gene 472:37-41.
3. Johannesen J, Foissac X, Kehrli P, Maixner M. 2012. Impact of Vector Dispersal and Host-Plant Fidelity on the Dissemination of an Emerging Plant Pathogen. Plos One 7.
4. Cvrkovic T, Jovic J, Mitrovic M, Krstic O, Tosevski I. 2014. Experimental and molecular evidence of *Reptalus panzeri* as a natural vector of bois noir. Plant Pathology 63:42-53.
5. Aryan A, Brader G, Mörter J, Pastar M, Riedle-Bauer M. 2014. An abundant '*Candidatus* Phytoplasma solani' tuf b strain is associated with grapevine, stinging nettle and *Hyalesthes obsoletus*. European Journal of Plant Pathology 140:213-227.
6. Murolo S, Romanazzi G. 2015. In-vineyard population structure of '*Candidatus* Phytoplasma solani' using multilocus sequence typing analysis. Infection Genetics and Evolution 31:221-230.
7. Quaglino F, Maghradze D, Casati P, Chkhaidze N, Lobjanidze M, Ravasio A, Passera A, Venturini G, Failla O, Bianco PA. 2016. Identification and characterization of new '*Candidatus* Phytoplasma solani' strains associated with Bois noir dsease in *Vitis vinifera* L. cultivars showing a range of symptom severity in Georgia, the Caucasus region. Plant Disease 100:904-915.
8. Balakishiyeva G, Bayramova J, Mammadov A, Salar P, Danet JL, Ember I, Verdin E, Foissac X, Huseynova I. 2018. Important genetic diversity of "*Candidatus* Phytoplasma solani" related strains associated with Bois noir grapevine yellows and planthoppers in Azerbaijan. European Journal of Plant Pathology 151:937-946.
